# Supplementary material for: A catalogue of biochemically diverse CRISPR-Cas9 orthologs
Source: Nat Commun. 2020 Nov 2;11:5512. doi: 10.1038/s41467-020-19344-1 (PMC7606464; doi:10.1038/s41467-020-19344-1)
Supplement: Supplementary file 3 — Description of Additional Supplementary Files [file 41467_2020_19344_MOESM3_ESM.pdf]

Description of Additional Supplementary Files

File Name: Supplementary Data 1
Description: Cas9 protein identity matrix.

File Name: Supplementary Data 2
Description: List of analysed Cas9 protein, gene, protein, and sgRNA sequences.

File Name: Supplementary Data 3
Description: Position frequency matrices (PFM) of Cas9 ortholog PAM recognition.

File Name: Supplementary Data 4
Description: List of PAM interaction domain sequences and cluster information.

File Name: Supplementary Data 5
Description: Target DNA cleavage patterns produced by Cas9 orthologs.

File Name: Supplementary Data 6
Description: List of Cas9 protein sequences used to construct the phylogenetic tree.

File Name: Supplementary Data 7
Description: Sequences of oligonucleotides and substrates used for this study.
