# Supplementary material for: A catalogue of biochemically diverse CRISPR-Cas9 orthologs
Source: Nat Commun. 2020 Nov 2;11:5512. doi: 10.1038/s41467-020-19344-1 (PMC7606464; doi:10.1038/s41467-020-19344-1)
Supplement: Supplementary file 11 — Reporting Summary [file 41467_2020_19344_MOESM11_ESM.pdf]

naturereseach

Corresponding author(s): Joshua K. Young, G. Brett Robb and Virginijus Siksnys

Last updated by author(s): Sep 23, 2020

Reporting Summary

Nature Research wishes to improve the reproducibility of the work that we publish. This form provides structure for consistency and transparency in reporting. For further information on Nature Research policies, see Authors & Referees and the Editorial Policy Checklist .

Statistics

For all statistical analyses, confirm that the following items are present in the figure legend, table legend, main text, or Methods section.

n/a	Confirmed	
x		The exact sample size (*n*) for each experimental group/condition, given as a discrete number and unit of measurement
x		A statement on whether measurements were taken from distinct samples or whether the same sample was measured repeatedly
	x	The statistical test(s) used AND whether they are one- or two-sided<br>*Only common tests should be described solely by name; describe more complex techniques in the Methods section.*
x		A description of all covariates tested
x		A description of any assumptions or corrections, such as tests of normality and adjustment for multiple comparisons
	x	A full description of the statistical parameters including central tendency (e.g. means) or other basic estimates (e.g. regression coefficient) AND variation (e.g. standard deviation) or associated estimates of uncertainty (e.g. confidence intervals)
	x	For null hypothesis testing, the test statistic (e.g. *F*, *t*, *r*) with confidence intervals, effect sizes, degrees of freedom and *P* value noted<br>*Give P values as exact values whenever suitable.*
x		For Bayesian analysis, information on the choice of priors and Markov chain Monte Carlo settings
x		For hierarchical and complex designs, identification of the appropriate level for tests and full reporting of outcomes
x		Estimates of effect sizes (e.g. Cohen's *d*, Pearson's *r*), indicating how they were calculated

Our web collection on statistics for biologists contains articles on many of the points above.

Software and code

Policy information about availability of computer code

Data collection	No software were used for data collection in this study.
Data analysis	PILER-CR 1.06, HMMER 3.2.1, MUSCLE 3.8.31 and MEGA7 10.0.5 were used for Cas9 identification and phylogenetic analysis. Secondary structures of tracrRNA were predicted with UNAFold 3.9 and visualised with VARNA 3-93. Computational tracrRNA analysis was performed using BLAST 2.7.3, CD-HIT 4.7, MAFFT 7.407, Infernal 1.1 and RNAalifold 2.4.5. We used Weblogo 2.8.2 for PAM visualization. MAFFT 7.407, PSI-BLAST 2.2.26, CD-HIT 4.6, CLANS 1.0, trimAL 1.2, IQtree 1.6.10 – were used for Computational analysis of Cas9 PAM interacting domains. DNA cleavage data were analyzed using Microsoft Excel 16.36 and GraphPad Prism 8.4. Custom scripts used to analyze cleavage patterns (https://github.com/greetsun/Cas-PAM-cleavage-analysis) and PAM preferences analysis ((https://github.com/cortevaCRISPR/Cas12f-InformaticsTools.git) have been deposited on github and a references provided in the manuscript.

For manuscripts utilizing custom algorithms or software that are central to the research but not yet described in published literature, software must be made available to editors/reviewers. We strongly encourage code deposition in a community repository (e.g. GitHub). See the Nature Research guidelines for submitting code & software for further information.

Data

Policy information about availability of data

All manuscripts must include a data availability statement. This statement should provide the following information, where applicable:

- Accession codes, unique identifiers, or web links for publicly available datasets
- A list of figures that have associated raw data
- A description of any restrictions on data availability

Raw deep sequencing data that support PAM and cleavage pattern determination for Cas9 orthologs have been deposited to the NCBI Sequence Read Archive Database with Accession numbers PRJNA622541 and PRJNA631559. All other relevant data are available from the corresponding authors on reasonable request. All protein sequences used for computational analysis are available in public databases (for example: UniRef100, MGnify, IMG/M, PDB), full list of accession numbers and sequences is provided in Supplementary Data 4 and 6.

Field-specific reporting

Please select the one below that is the best fit for your research. If you are not sure, read the appropriate sections before making your selection.

☒ Life sciences ☐ Behavioural & social sciences ☐ Ecological, evolutionary & environmental sciences

For a reference copy of the document with all sections, see nature.com/documents/nr-reporting-summary-flat.pdf

Life sciences study design

All studies must disclose on these points even when the disclosure is negative.

Sample size	Our studies did not use sampling
Data exclusions	No data were excluded
Replication	All quantitative analyses were replicated at least three times independently, all attempts to replicate were successful and gave similar results
Randomization	Our study did not involve grouping of experimental subjects, thus randomization was not warranted
Blinding	Our experimental design did not use subjective scoring by researchers for any of the data reported for which blinding would have impacted the collection or interpretation of the data

Reporting for specific materials, systems and methods

We require information from authors about some types of materials, experimental systems and methods used in many studies. Here, indicate whether each material, system or method listed is relevant to your study. If you are not sure if a list item applies to your research, read the appropriate section before selecting a response.

Materials & experimental systems

n/a	Involved in the study	
☒	☐	Antibodies
☒	☐	Eukaryotic cell lines
☒	☐	Palaeontology
☒	☐	Animals and other organisms
☒	☐	Human research participants
☒	☐	Clinical data

Methods

n/a	Involved in the study	
☒	☐	ChIP-seq
☒	☐	Flow cytometry
☒	☐	MRI-based neuroimaging
